# 

**Published:** 1885-10

**Authors:** 


### THE CHICAGO MEDICAL JOURNAL AND EXAMINER

EDITORS:

S. J. JONES, M.D., LL.D.

W. W. JAGGARD, A.M., M.D. HAROLD N. MOYER, M.D.

CHICAGO:

PUBLISHED MONTHLY BY THE CHICAGO MEDICAL PRESS ASSOCIATION

240 WABASH AVENUE.

CLARK & LONGLEY PRINTERS, 163 AND 165 DEARBORN STREET, CHICAGO.

